# The impact of intermediate product imports on industrial pollution emissions: Evidence from 30 industries in China

**DOI:** 10.1371/journal.pone.0292347

**Published:** 2023-10-04

**Authors:** Lu Wan, Yuling Mao, Yizhong Fu, Xiya Wan

**Affiliations:** School of Economics and Management, Beijing Forestry University, Beijing, China; East China Normal University, CHINA

## Abstract

Open and sustainable development is the theme that underpins a country’s high-quality economic development. This study uses GMM regression, mediation effect test to conduct empirical tests based on the panel data of China’s industrial sectors from 2003 to 2015 to analyze the internal mechanism of the impact of intermediate product imports on China’s industrial pollution emissions. The results show that (1) Intermediate product imports can significantly promote the emission reduction of industrial wastes, including wastewater, waste gas and solid waste. (2) Considering the differences in the level of pollution intensity, this paper classified the sample and found the impact is heterogeneous that for the heavily, moderately, lightly polluted industries, intermediate product imports have different negative impacts on their pollution emissions. (3) Intermediate products imports reduce industrial pollution emissions through import competition effect, variety effect and technology spillover effect, and all of them play a partial mediating role.

## Introduction

Since the reform and opening up, China has actively integrated into the global production system and vigorously promoted the liberalization of intermediate trade, which has led to a significant improvement in production efficiency and rapid economic growth. However, due to its extensive economic growth mode and continuously expanding industrialization scale, the negative impact on the environment is gradually becoming prominent. In order to positively respond to global climate change and push forward high-quality and sustainable development of the Chinese economy, China proposed a specific implementation plan for environmental and ecological protection in 2020, resolutely curbing the blind development of high energy consumption and high emission projects. It can be seen that China attaches great importance to environmental pollution issues and has been seeking a green path for harmonious economic development and environmental protection.

China entered a new round of rapid import trade liberalization after being admitted into the WTO at the end of 2001. From 2003 to 2021, the scale of China’s foreign trade imports increased from 0.41 trillion US dollars to 2.69 trillion US dollars, accounting for a proportion of global imports from 4.4% to 11.9%. Meanwhile, according to WTO, the import volume of intermediate goods was 1676 billion US dollars, 65% and 176% higher than the United States (second place) and Germany (third place) respectively. China has become the largest country in intermediate goods trade. With the reform of the liberalization system of intermediate goods trade, the structure of intermediate goods trade is being optimized faster, reflected in the significant growth of key materials, components, and other intermediate goods in the manufacturing supply chain. In this context, the import of intermediate goods can help to resolve the current structural contradictions on China’s supply side, encourage enterprises to allocate resources globally, accelerate the transformation of production methods, and thus become a new path for carbon reduction and pollution reduction.

Regarding trade and environment, numerous studies have constructed theoretical and empirical models of the impact of international trade on environmental quality and conducted further analysis. It is worth mentioning that Johnson et al. [1] found that once the intermediate products trade accounted for more than 66.7% of total trade, the impact would be equivalent to that of the final products trade. Through a comparative study, He and Hertwich [2] discovered that intermediate product trade can result in implicit carbon emissions equivalent to final product trade. From the perspective of import structure, the proportion of intermediate goods imported by China reached 67.61% in 1995, and even increased to 75.71% in 2020 [3], demonstrating that its impact on environmental quality cannot be overstated. Under such a trade scale, what impact will intermediate product imports have on China’s industrial pollution emissions? Will industrial sectors with different levels of pollution be affected differently? What is its impact mechanism? In order to answer the above questions, this study selects 30 industrial sectors in China, deeply explores the impact of intermediate import trade on the pollution emissions of the industrial sectors and further examines the internal mechanism.

Compared to existing research, the contributions of our work are mainly reflected in the following aspects. First, for the research perspective, exploring the role of import trade in industrial pollution emissions based on intermediate goods trade fills the research gap in the intersection of intermediate goods imports and the environment, and provides new empirical evidence for the reduction of carbon emissions under open economic conditions; Second, in terms of research subjects, we utilized data from the Chinese industrial sector level and used GMM regression to examine the impact and mechanism of intermediate product imports on pollution emissions. Considering the heterogeneity of the industrial sector, we then divides it into three sub samples based on the intensity of pollution emissions for detailed exploration, targeting the largest country in intermediate trade as a reference for improving green development in developing countries; Third, as for research content, the competition effect, variety effect, and technology spillover effect of intermediate goods imports are specifically measured to test their indirect mechanism, further supplementing and improving the possible paths of intermediate goods imports affecting industrial pollution, providing important insights for achieving pollution reduction from international trade.

## Literature review

Trade and environmental issues have always been a hot topic of concern for academia, so a wealth of literature has been accumulated. It is possible to categorize the research that are closely linked to this paper into two groups: one is literature discussing the relationship between intermediate import trade and environmental pollution. By researching the relationship between trade and environmental contamination, some academics have reached conflicting conclusions, that is, import trade may inhibit environmental pollution or exacerbate pollution; The other is literature that discusses the effects of intermediate goods imports. Most literature found that intermediate goods imports improve total factor productivity, export product quality, and technological level of enterprises through competition, variety and technology spillover effect, which are important channels for developing countries to progress.

### The impact of import trade on the environment

In the development of trade environment theory, Grossman and Krueger [4] pioneered the decomposition of the impact of trade on the environment into scale effect, composition effect, and technique effect, and empirically studied the impact of the North American Free Trade Agreement (NAFTA) on the environment. Based on this analytical framework, free trade will improve environmental quality to some extent, and when income reaches a certain level, the negative impact of scale effect will be surpassed by the positive impact of composition and technique effect. This income level is the critical value represented at the inflection point of the inverted U-shaped Environmental Kuznets Curve (EKC), which reflects the relationship between per capita income and the degree of environmental degradation. The classic theory is still inspiring a lot of empirical research to this day. The results of Murthy and Gambhir [5] confirmed that India presents an N-type EKC, proving that India has indeed become a pollution paradise for developed countries in the process of economic development. But while Awaworyi [6] used Australia as a sample for analysis, the results obtained were consistent with the inverted U-shape of traditional EKC.

With the further deepening of international trade, the depth and breadth of research in this field have also been expanded. From the perspective of import trade, Dietzenbacher and Mukhopadhyay [7] investigated the impact of trade activities between countries on environmental pollution and found that although India’s import and export trade generally exacerbates pollution, the pollution reduction caused by import trade is less than the pollution increase caused by export trade. Yao et al. [8] studied the energy efficiency of 36 countries worldwide and found that the impact of export value added on energy efficiency is much greater than that of import value added. Chen et al. [9] estimated the energy intensity of 30 provinces in China from 2005 to 2018 and explored the impact of trade openness and economic growth on China’s energy intensity. They concluded that the role of foreign trade in energy intensity is mainly attributed to export channels, whereas the role of import channels can be ignored. This may be the reason why import trade has not received sufficient attention in relevant research. However, with the development of emerging economies and the extension of global production networks, intermediate trade has come to the foreground owing to its pivotal position in import trade. Through energy use efficiency, Michele and Ketterer [10] indirectly measured the environmental performance of enterprises and found that the energy use efficiency of intermediate import enterprises was higher than that of enterprises that had never imported before in Indonesia. Zhang [11] investigated the relationship between intermediate product trade and pollution in the context of endogenous environmental policies. The results showed that developed countries may reduce pollution at the cost of more pollution from developing countries. In other words, the pollution paradise hypothesis also exists in intermediate product trade, which may cause environmental degradation in developing countries. It reflected the academic circle has not yet reached a consistent conclusion on the environmental benefits of intermediate product imports, so relevant research urgently needs to be supplemented.

### The effects of intermediate product imports

When studying import trade, intermediate goods, serving as carriers of new knowledge and technology, became an important form of technology diffusion and transfer [12]. As a form of materialized technology spillover, it also promotes technological progress and enterprise innovation in importing countries. Grossman and Helpman [13] were the first to introduce the general equilibrium model to analyze the relationship between trade, growth, and technological progress in an open economy, and discussed how trade in intermediate and final goods affects long-term economic growth. Based on this, Coe and Helpman [14] empirically examined the impact of import trade on international technology spillovers and total factor productivity growth. Their method of weighting the R&D of trading partner countries with import share has become a common practice in subsequent research on import trade as an international technology spillover channel. Schmit [15] summarized the intermediate import effect as the expansion of intermediate import scale, improvement of intermediate import quality, and spillover of intermediate import technology, which is the learning effect in imports. Amiti et al. [16] examined the productivity benefits of Indonesia’s import liberalization from the perspective of tariff reduction, believing that reducing tariffs on final products can introduce more intense import competition to improve productivity, while intermediate imports improve productivity through learning effects. Halpern et al. [17] studied the degree to which total factor productivity is affected by intermediate goods import trade. They pointed out that in the context of trade openness and globalization, intermediate goods import contributes about 30% of the driving force to the growth of total factor productivity.

In addition to the technology spillover effect caused by the embedded technology level of exporting countries in imported intermediate goods, Feenstra [18] first adopted the measurement method of the net variety change index of imported products, estimated the welfare effect brought by the substitution elasticity between the change in the number of imported varieties and the share of import costs. It was discovered that an increase in the variety of imported products would lower the import price index and improve trade conditions. And the technical method of measuring changes in import varieties through micro trade data laid the foundation for scholars to examine the relationship between imports and corporate behavior from diversified import products. Subsequently, Goldberg et al. [19] applied this method to measure changes in intermediate import types, which drew that an increase in intermediate import types would promote the improvement of product quality and innovation capabilities of enterprises. This conclusion has also been supported by Liu and Qiu [20] and Chen and Zhang [21]. Based on panel data from prefecture level cities in China, Xiang et al. [22] also asserted that intermediate goods imports contribute to improving urban energy efficiency through technology spillover effect and diversification of intermediate goods types. Besides, Melitz [23] believes that the domestic market competition brought about by imports enhances the price elasticity of demand for enterprises, which can lead to lower productivity enterprises exiting the market and reduce product prices and markup rates.

In summary, we can see the shortcomings of existing research: First, although a large number of literature has focused on the relationship between trade and environmental pollution, most of them show solicitude for exports and final products. This paper studies from the perspective of intermediate goods imports, filling the gap in this field. Second, research on the practical impact of intermediate goods import trade mainly concentrates on the technology spillover effects on enterprise productivity and export product quality. There is little literature to test whether these mechanisms are the channels through which intermediate goods import affects industrial pollution emissions. Third, existing literature are mostly based on the macro and micro enterprise levels, so there is a lack of research on the heterogeneity of industrial sectors. We divide the industrial sector into three samples based on pollution emission intensity to explore the impact of intermediate goods imports on heterogeneous industrial pollution emissions. This not only adds empirical evidence from the world’s largest developing country to the existing research on intermediate goods imports and environment, but also provides a new perspective for evaluating the effectiveness of China’s intermediate trade liberalization reform afterwards.

## Methodology

Through a review of existing research, we summarize the impact pathways of intermediate product imports on industrial pollution emissions as competition effect, variety effect, and technology spillover effect. In the study on the mechanisms that affect environmental pollution, literature mostly considered technical factors into the impact pathways of environmental pollution for empirical testing, and drew conclusions that can affect environmental pollution through technical pathways. We combine the three types of import effects of intermediate goods and uses technological progress as a link to analyze the impact path between intermediate goods imports and industrial pollution emissions.

The rich variety and wide source of intermediate products imported by enterprises indicate that the market for imported products is filled with competition everywhere. Firstly, for intermediate suppliers, the entry of low-cost and high-quality intermediate products into the domestic market will put pressure on domestic manufacturers of similar intermediate products. Downstream enterprises will be more inclined to use imported intermediate products with low cost, which will squeeze the profits of domestic intermediate suppliers. The impact of high-quality products from external sources will result in the elimination of products that do not have quality advantages produced by enterprises themselves from the production process, freeing up various means of production, including machinery and equipment, labor, capital, and other means of production. This production model of reducing peripheral products and focusing on core products can not only enable enterprises to concentrate resources on the production of core products [3], but also increase one’s own research and development investment to promote technological progress and develop new and more advantageous intermediate products [21]. Secondly, for the final product manufacturer, when the price of the intermediate products required increases due to the inclusion of advanced foreign technological elements, the production cost and price of the final product will also increase for the manufacturer who produces the corresponding final product, resulting in the manufacturer losing its price competitive advantage in the international market, so as to forcing enterprises to change their operational management methods or improve their production technology to reduce long-term production costs and regain market position [24, 25]. Based on this, we conclude that:

Hypothesis 1: Intermediate product imports affect technological progress through competitive effect

One phenomenon that cannot be ignored is that the diversification of intermediate goods imports has become a trend. Due to the incomplete substitution of technology and knowledge internalized by different products or product types, the technological progress brought about by the increase in diversity of imported intermediate products will expand exponentially. This paper summarizes the impact of the increase in the varieties of imported intermediate goods on technological progress into three channels. The first is the learning effect. Within the increase in the types of imported intermediate goods, enterprises have come into contact with and use more varieties of key spare parts inputs and advanced production equipment capital goods. High tech intermediate products from developed countries contain advanced processes that can be internalized by enterprises for learning, thereby improving their technological level and innovation capabilities [15]; The second is the complementary effect, which can easily produce a "the whole is more than the sum of parts" effect by combining different products or types of intermediate products (imported intermediate products with imported intermediate products, imported intermediate products with domestic intermediate products) [26]. Likewise, considering the large number of intermediate product assembly activities of Chinese enterprises, by importing intermediate products and combining them with the mutual matching model, if enterprises can integrate their own research and development efforts, the process of innovating products will be simpler and faster [27]; The third is the R&D incentive effect. The increase in import varieties enhances the global resource allocation efficiency of enterprises, improves production efficiency, price markup, and profit income, reduces financial constraints, and encourages enterprises to invest more available funds in long-term independent innovation activities [28]. Since the higher level of quality or technical components in new products, the expected revenue of enterprises for new products has been increased, which further stimulates the research and development behavior of enterprises. Based on this, we conclude that:

Hypothesis 2: Intermediate product imports affect technological progress through variety effect.

As early as the 1980s, some scholars proposed that the spillover effect of technology in import trade is exerted through both horizontal and vertical perspectives, so we also analyzed it according to this logic. Firstly, the horizontal spillover effect mainly refers to the technological connection between intermediate import enterprises and domestic intermediate producers. On the one hand, a large number of foreign intermediate products are integrated into the domestic market, causing import competitive enterprises in the same industry to face fierce market competition. In order to occupy market share and continue to survive and develop, these import enterprises will absorb advanced technologies contained in imported intermediate products through competition and imitation [16]. On the other hand, market demand pressure forces unskilled workers to continuously improve their own quality and knowledge skills, gradually adapting to high-end production and research and development [29], stimulating the motivation of domestic suppliers for technological innovation, thereby driving the overall technological upgrading of the country and improving the level of technological innovation of domestic enterprises. Secondly, vertical spillover effect refers to the transmission of advanced production factors contained in imported intermediate goods through the production chain. If the technology of a certain link or industry in the industrial chain improves, other industries in other links will improve their production technology to match the technology, thus forming a vertical spillover of technology. Especially in China’s processing trade, the technology in imported intermediate goods will be transmitted to manufacturers engaged in processing trade, promoting their technological progress, which will inevitably have a demand for technological innovation in their upstream and downstream industries. Therefore, the technological innovation of enterprises has a great promoting effect on the technological innovation of related enterprises. Based on this, we conclude that:

Hypothesis 3: Intermediate product imports affect technological progress through technological spillover effect.

Firstly, the technological progress brought by the import of intermediate products will lead to improvements in production efficiency. Technological progress has improved the utilization rate of resources or raw materials in the production process, enabling the same resources or raw materials to produce more products compared to traditional production technologies; Secondly, technological progress will include innovation in green technologies to reduce pollution emissions during product production. In addition, technological progress promotes pollution reduction through green research and development of products, recycling and comprehensive utilization of excess resources or raw materials. The advanced technology of greening and cleaning is used to monitor the entire production process of enterprise products, and to prevent and control pollution emissions in advance, which is more efficient than post prevention and control in reducing pollution emissions. In terms of pollution control, the discharge of industrial pollutants requires enterprises to solve it through technological means. Improving the level of pollution control technology can effectively reduce the discharge of industrial three wastes; Thirdly, technological progress can improve product production and pollution control technologies, achieving the production of green products. The widespread emergence of green products in the market can awaken people’s environmental awareness, making consumers more inclined to choose green concept products during the consumption process, and enhancing people’s understanding of sustainable development. It can be seen that technological progress is an important channel and necessary path for green development. Based on this, we conclude that:

Hypothesis 4: The import of intermediate goods affects technological progress through the above three import effects, thereby improving enterprise production efficiency and enhancing green technology innovation, green product research and development, which are carried out to promote pollution reduction.

## Empirical model and variables

### The empirical model

It is universally acknowledged that pollutant emissions will be influenced by previous emissions. Hence, the first pollutant lag period is introduced in order to build a regression model for the dynamic impact of intermediate product imports on industrial pollution emission. Furthermore, Grossman and Krueger [4] asserts that environmental pollution will worsen with economic growth at first, but that once the level of economic development reaches a certain value, environmental pollution will begin to relieve, the entire process taking the shape of an inverted U. So, in accordance with Antweiler et al. [30], the primary and secondary variables of economic development level are thus added to the equation. The following is the empirical model:

$${lnFS}_{i,t}=\alpha_{0}+\alpha_{1}ln{FS}_{i,t-1}+\alpha_{2}ln{IMIN}_{i,t}+\alpha_{3}{FDI}_{i,t}+\alpha_{4}ln{SIZE}_{i,t}+\alpha_{5}ln{FSERS}_{i,t}+\alpha_{6}ln{GDP}_{t}+{\alpha_{7}{ln}^{2}{GDP}_{t}+\alpha }_{8}ln{RD}_{i,t}+\varepsilon_{i,t}$$ (1)

$${lnFQ}_{i,t}=\beta_{0}+\beta_{1}ln{FQ}_{i,t-1}+\beta_{2}ln{IMIN}_{i,t}+\beta_{3}{FDI}_{i,t}+\beta_{4}ln{SIZE}_{i,t}+\beta_{5}lnFQ{ERS}_{i,t}+\beta_{6}ln{GDP}_{t}+\beta_{7}{ln}^{2}{GDP}_{t}+\beta_{8}ln{RD}_{i,t}+\varepsilon_{i,t}$$ (2)

$${lnFG}_{i,t}=\gamma_{0}+\gamma_{1}ln{FG}_{i,t-1}+\gamma_{2}ln{IMIN}_{i,t}+\gamma_{3}{FDI}_{i,t}+\gamma_{4}ln{SIZE}_{i,t}+\gamma_{5}ln{FGERS}_{i,t}+\gamma_{6}ln{GDP}_{t}+\gamma_{7}{ln}^{2}{GDP}_{t}+\gamma_{8}ln{RD}_{i,t}+\varepsilon_{i,t}$$ (3)

where i represents industrial sectors, t the years from 2003 to 2015, and α_j_, β_j_, γ_j_ to represent the coefficients to be estimated. FS_i,t_, FQ_i,t_ and FG_i,t_ respectively indicate the industrial emissions of wastewater, waste gas emission and solid waste. IMIN_i,t_ refers to the import trade volume of intermediate goods. FDI_i,t_ is the foreign direct investment introduced by the industry. SIZE_i,t_ is the scale of the industry. FQERS_i,t_, FSERS_i,t_ and FGERS_i,t_ respectively indicate the environmental control level of the corresponding waste. lnGDP_t_ and ln^2^GDP_t_ are the primary term and quadratic term of the economic development level after taking the logarithm. RD_i,t_ refers to the R&D investment of industry.

Combined with the analysis of the theoretical mechanism, this paper further examines the indirect effect of intermediate product imports on pollution emissions of China’s industrial sectors. Similar to the practice of Cheng [31] and Gao [32], a mediation effect model is further constructed with the mediating variables of the import variety effect index and technology spillover effect index:

$${DI}_{i,t}=\beta_{0}+\beta_{1}{DI}_{i,t-1}+\beta_{2}ln{IMIN}_{i,t}+\beta_{3}{FDI}_{i,t}+\beta_{4}ln{SIZE}_{i,t}+\beta_{5}ln{ERS}_{i,t}+\beta_{6}ln{GDP}_{t}+\beta_{7}{ln}^{2}{GDP}_{t}+\beta_{8}ln{RD}_{i,t}+\varepsilon_{i,t}$$ (4)

$${lnEVI}_{ijt}=\gamma_{0}+\gamma_{1}ln{EVI}_{i,t-1}+\gamma_{2}{DI}_{i,t}+\gamma_{3}ln{IMIN}_{i,t}+\gamma_{4}{FDI}_{i,t}+\gamma_{5}ln{SIZE}_{i,t}+\gamma_{6}ln{ERS}_{i,t}+\gamma_{7}ln{GDP}_{t}+\gamma_{7}{ln}^{2}{GDP}_{t}+\gamma_{8}ln{RD}_{i,t}+\varepsilon_{i,t}$$ (5)

where DI_i,t_ refers to the variety effect index of imported intermediates, which can reflect the changes in the varieties of imported intermediates. EVI_ijt_ represents the emission or production of pollutants of variety j, including industrial wastewater, waste gas and solid waste. The technology spillover effect indicator is TEC_i,t_, used as another mediating variable to establish a mediation effect model similar to Formulas (4) and (5).

### Variables

#### Dependent variable

In previous studies, the pollution emission, is generally selected as a variable to measure environmental pollution, mainly including SO_2,_ CO_2_, industrial waste gas, wastewater, and solid waste. However, this paper draws on the practices of many scholars and selects industrial wastewater, industrial waste gas and industrial solid waste emissions as the dependent variables [33].

#### Independent variable

This paper directly uses the import scale of intermediate goods as the core independent variable, that is, the trade volume of intermediate goods imported from various countries by various industries [34]. Combined with the above analysis and elaboration, it is believed that the import of intermediate goods has a positive impact on the industrial pollution reduction.

#### Control variables

(1) Foreign Direct Investment (FDI). This paper measures the proportion of foreign investment in various industries and investment enterprises in Hong Kong, Macao and Taiwan in total assets of various industries. (2) Environmental Regulation (ERS). The proportion of waste gas and wastewater treatment operating costs in the industrial sales output value is used to measure the industrial wastewater environmental regulation (FSERS) and industrial waste gas environmental regulation (FQERS). The environmental regulation of industrial solid waste (FGERS) is directly measured by the amount of solid waste disposal. They are used as proxy variables for the effect of environmental regulation. Additionally, due to the differences in the production structure of various industries, the gross industrial output value of the industry needs to be adjusted. (3) Industry Size (SIZE). It is expressed by gross industrial output value. (4) R&D investment (RD). Use R&D intensity (RD expenditure/main business income) as an indicator to measure industry R&D investment. (5) The level of economic development (GDP). A country’s economic development level is measured by per capita GDP.

#### Mediating variables

(1) Variety effect index

This paper uses the methods of Feenstra [18] to construct a diversity index that reflects the variety of imported intermediate:

$${DI}_{it}=\frac{\sum_{d\in D}T_{dt-1}}{\sum_{d\in D_{t-1}}T_{dt-1}}/\frac{\sum_{d\in D}T_{dt}}{\sum_{d\in D_{t}}T_{dt}}$$ (6)

where, d is the variety of imported intermediates, D_t_ is the total of all varieties of intermediates imported in period t, D_t−1_ is the collection of all varieties of intermediates imported in period t, and D includes period t and t-1. T_dt_ and T_dt−1_ represents the import value of the intermediate goods imported by variety d in period t and period t-1. If it is 1, it means that DT_it_ there is no change in the varieties of imported intermediates in period t and t-1. If DI_it_ = 1, it means that there is no change in the types of intermediate goods imported in phase t and t-1. If it>1, it means that the varieties of imported intermediate goods in phase t increase; otherwise, it means that the decrease.

(2) Technology spillover effect index

Based on the approach of Lichtenberg and Pottelsberghe [33], the following calculation formula is constructed to measure the effect index of technology spillover from intermediate product imports:

$${TEC}_{it}=\sum_{q}\frac{{iminput}_{iqt}}{{GDP}_{qt}}\times S_{qt}^{d}$$ (7)

where TEC_it_ is the R&D spillover stock obtained by industry i through intermediate product imports in year t. iminput_iqt_ is the total amount of intermediate goods imported. GDP_qt_ is the gross domestic product of country q in year t. $S_{qt}^{d}$ represents the domestic R&D stock, using the perpetual inventory method $S_{qt}^{d}=(1-5\%)S_{qt-1}^{d}+{RD}_{jt}$. Among them, 5% is the depreciation rate of R&D capital. RD_qt_ is the R&D expenditure.

The specific variable descriptions and definitions are shown in Table 1.

**Table 1:** Variable description and definition.

Variable type	Variable	Variable description	Symbol
Dependent variable	Industrial wastewater	Annual emission of industrial wastewater by industry	FS
	Industrial waste gas	Annual emissions of industrial waste gas by industry	FQ
	Industrial solid waste	Emissions of industrial solid waste by industry	FG
Independent variables	Intermediate goods import trade volume	The total trade volume of intermediate goods imported from various countries by industry each year	IMIN
Control variables	Foreign direct investment	The proportion of total assets of foreign-invested enterprises in various industries and investment enterprises in Hong Kong, Macao and Taiwan regions in the total assets of various industries	FDI
	Industrial wastewater environmental regulation	Proportion of industrial wastewater treatment operating costs in various industries to industrial sales output value	FSER
	Industrial waste gas environmental regulation	Proportion of industrial waste gas treatment operating costs in various industries to industrial sales output value	FQER
	Industrial solid waste environmental regulation	Disposal volume of industrial solid waste by industry	FGER
	Industry size	Industrial sales output value of various industries	SIZE
	R&D investment	Proportion of R&D expenditure in each industry to main business income	RD
	The level of economic development	GDP per capita	GDP

### Data

Due to the lack of China Environmental Statistics Yearbook in 2002, and since 2016, the China Environmental Statistical Yearbook has not counted the emission of industrial wastewater, industrial waste gas and industrial solid waste in each industry, this paper uses the data of China’s industry from 2003 to 2015. The data of industrial wastewater emission, industrial waste gas emission and industrial solid waste emission are mainly obtained from the China Environmental Yearbook and China Environmental Statistical Yearbook from 2004 to 2016. The trade volume of intermediate goods imported by various industries is obtained from the WITS database. GDP per capita data is from the World Bank database. The data of environmental regulation, foreign direct investment and industry scale are from China Statistical Yearbook and China Industrial Economic Yearbook from 2004 to 2016; The R&D investment data of various industries are from the China Science and Technology Statistics Yearbook from 2004 to 2016. In order to eliminate the impact of exchange rate changes on the data, the annual average exchange rate of the year is used to convert the import trade volume of intermediate goods and GDP per capita into statistical data denominated in RMB. Moreover, the natural logarithm treatment of industrial wastewater emission, industrial waste gas emission, industrial solid waste emission, intermediate product imports, industry scale, industry R&D investment and per capita GDP is carried out.

## Characteristic facts

This section analyzes the current situation of China’s industrial intermediate import trade from both macro and micro aspects. According to China’s classification of industrial sectors and SITC Rev3 and BEC, with the reference of the correspondence between industry and intermediate products in Sheng [34], SITC codes of imported intermediate goods in 30 industrial sectors in China were screened. The SITC code is used to obtain product data from the WITS database, and the import of intermediate goods in the industrial sectors is analyzed from the import scale, the structure of imported goods and the structure of the import source country.

### The value of intermediate product imports

**Fig 1:**
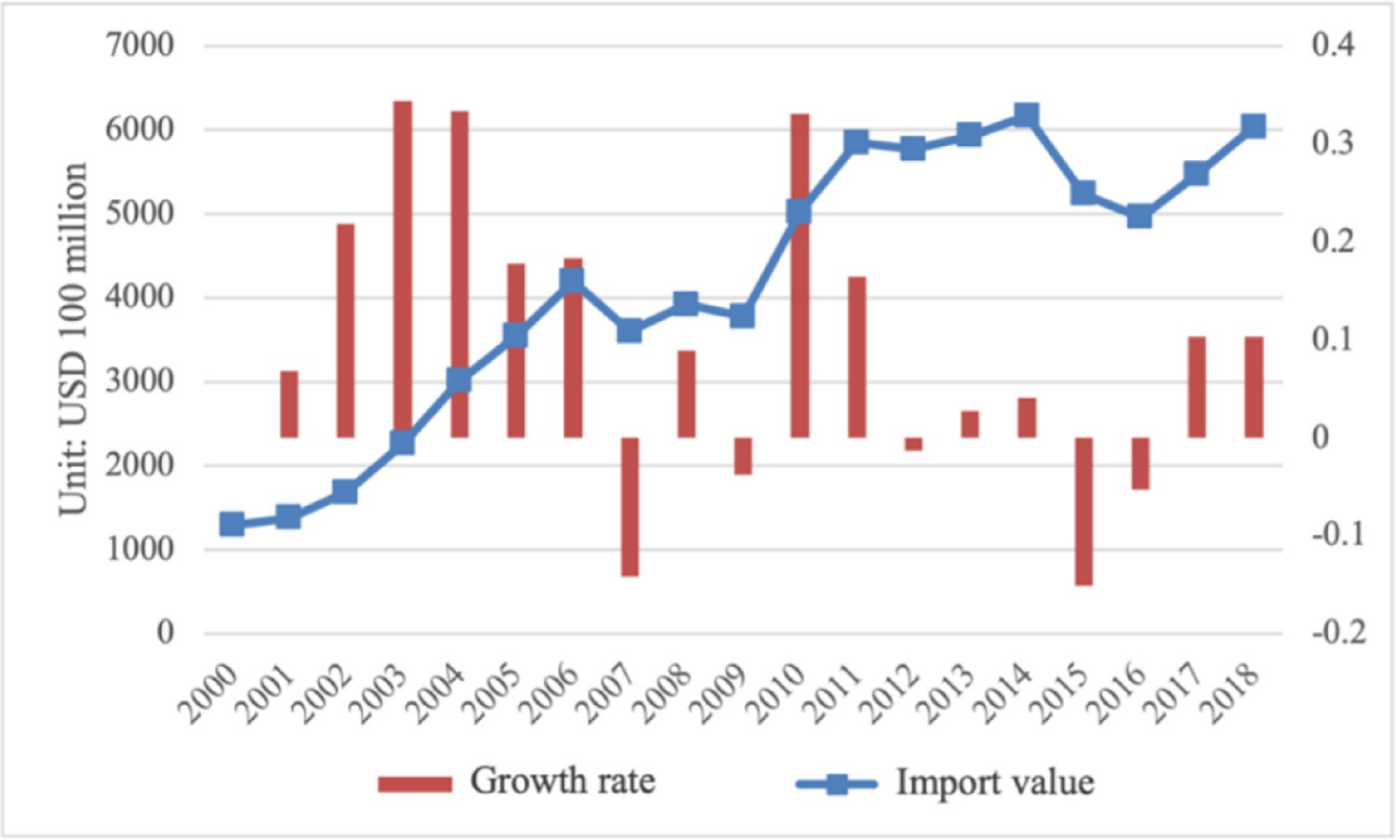
The import value and growth rate of intermediate products in the industrial sector from 2000 to 2018. Source: data were obtained from the WITS database.

The overall growth rate of intermediate product imports in the industrial sector showed a downward trend. Fig 1 shows the import value and growth rate of intermediate goods in the industrial sector from 2000 to 2018. Specifically, in 2000, the import value of intermediate goods in the industrial sectors was 170.6878 billion US dollars, and in 2018, it increased to 1,612.195 billion US dollars, which is about 9.45 times that of 2000. During different periods, there are different development characteristics. Due to its successful entry into the WTO in 2001, tariffs have dropped and the scale of intermediate product imports has risen sharply. Its trade growth rate was as high as 30% in 2003, the highest growth rate in recent years. However, because of the financial crisis in 2018, the important role of manufacturing in the national economic development was re recognized by developed economies, so a series of preferential policies and measures were issued to support the return of manufacturing enterprises. And in light of the implementation of these preferential policies and measures, manufacturing multinational companies in developed countries will gradually withdraw their enterprises or factories from China. As demand decreases, the import of intermediate goods will also decrease. Compared with some emerging developing economies, China’s demographic dividend and cost comparative advantages are gradually disappearing. Therefore, a large number of multinational companies will choose countries with advantages in demographic dividend and cost to move into their factories. By 2010, the economy had recovered, and the import trade volume of intermediate goods had grown rapidly, with a trade growth rate of more than 30%, and the growth rate gradually slowed down in the following years. In 2014, due to the impact of commodity prices, the value of trade imports fell, and since 2016, the value of trade has started to rise again. In general, from 2000 to 2018, the import volume of intermediate products in the industrial sectors was volatile.

### The structure of intermediate product imports

**Fig 2:**
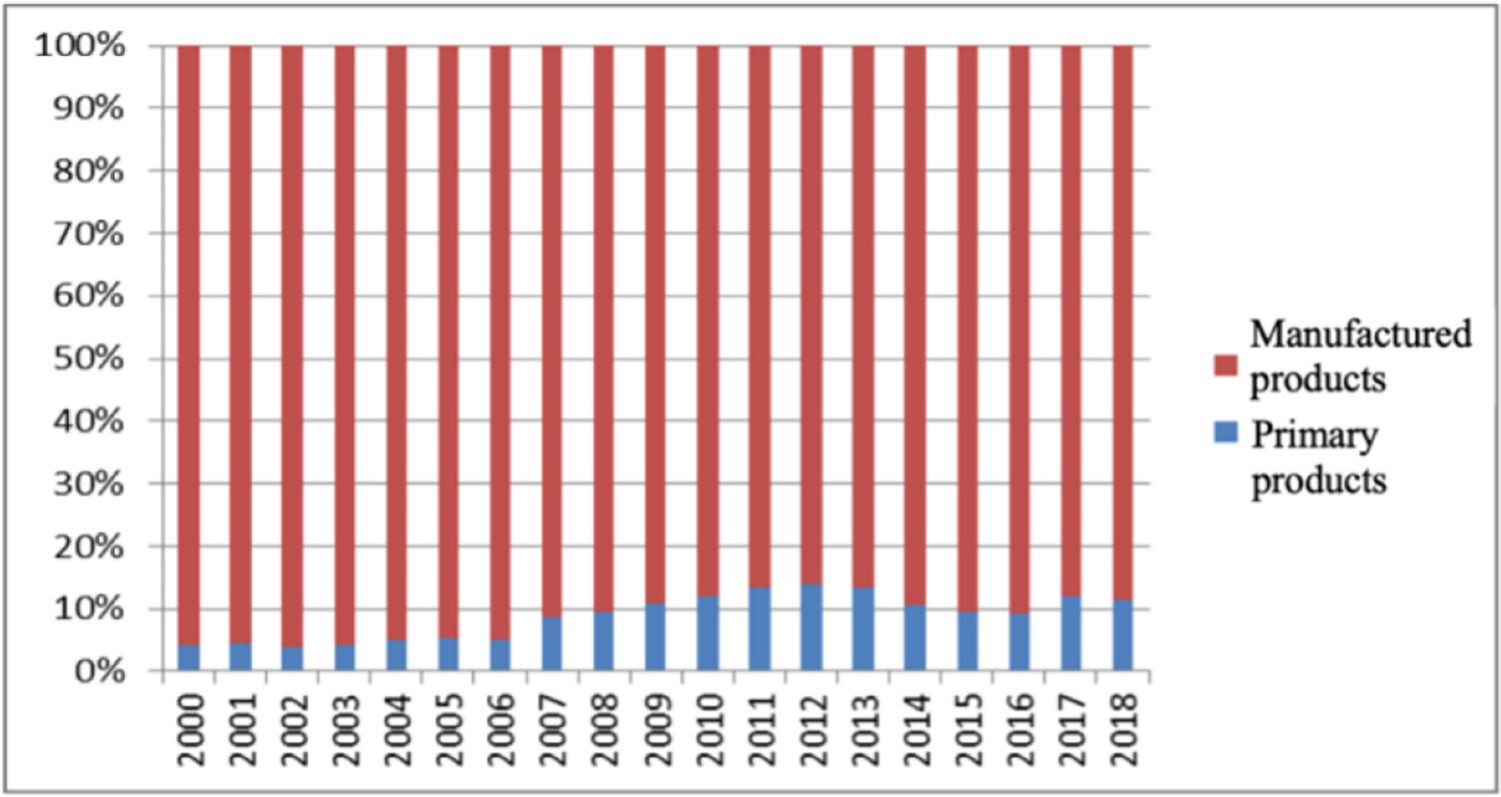
Proportion of imports of intermediate products in preliminary classification. Source: data were obtained from the WITS database.

Drawing on the practice of Wei [35], intermediate goods are divided into two categories: primary products and manufactured products. Fig 2 shows the proportion of primary products and manufactured products in the imported intermediate products of the industrial sectors from 2000 to 2018. By observing the product import proportion of intermediate products, from 2000 to 2018, manufactured products have always been the largest import variety of intermediate products in China’s industrial sectors. From 2000 to 2008, the import of primary products accounted for less than 10% of intermediate product imports in the industry, but the proportion was increasing. Since 2009, the proportion of primary products has remained around 10%, and reached the highest in recent years in 2012. Primary products mainly include resource-based products. Since the domestic supply of resource-based products is insufficient to meet the demand, it is necessary to increase the import of resource-based intermediate products from abroad.

According to Wei [35] on the structure of intermediate products, this paper divides intermediate products into the following four categories again. The specific classification is shown in Table 2.

**Table 2:** Reclassification of intermediate products.

Product category	Reclassification	Representative product
Primary products	Resource products	Ore, food, meat and vegetables
Manufactured products	Low-tech manufactured products	Wood products, paper and cardboard, mineral resources, textiles, toys
	Chinese technology industrial products	Chemicals, machinery
	High-tech industrial products	Medicine and Power Generation Equipment

**Fig 3:**
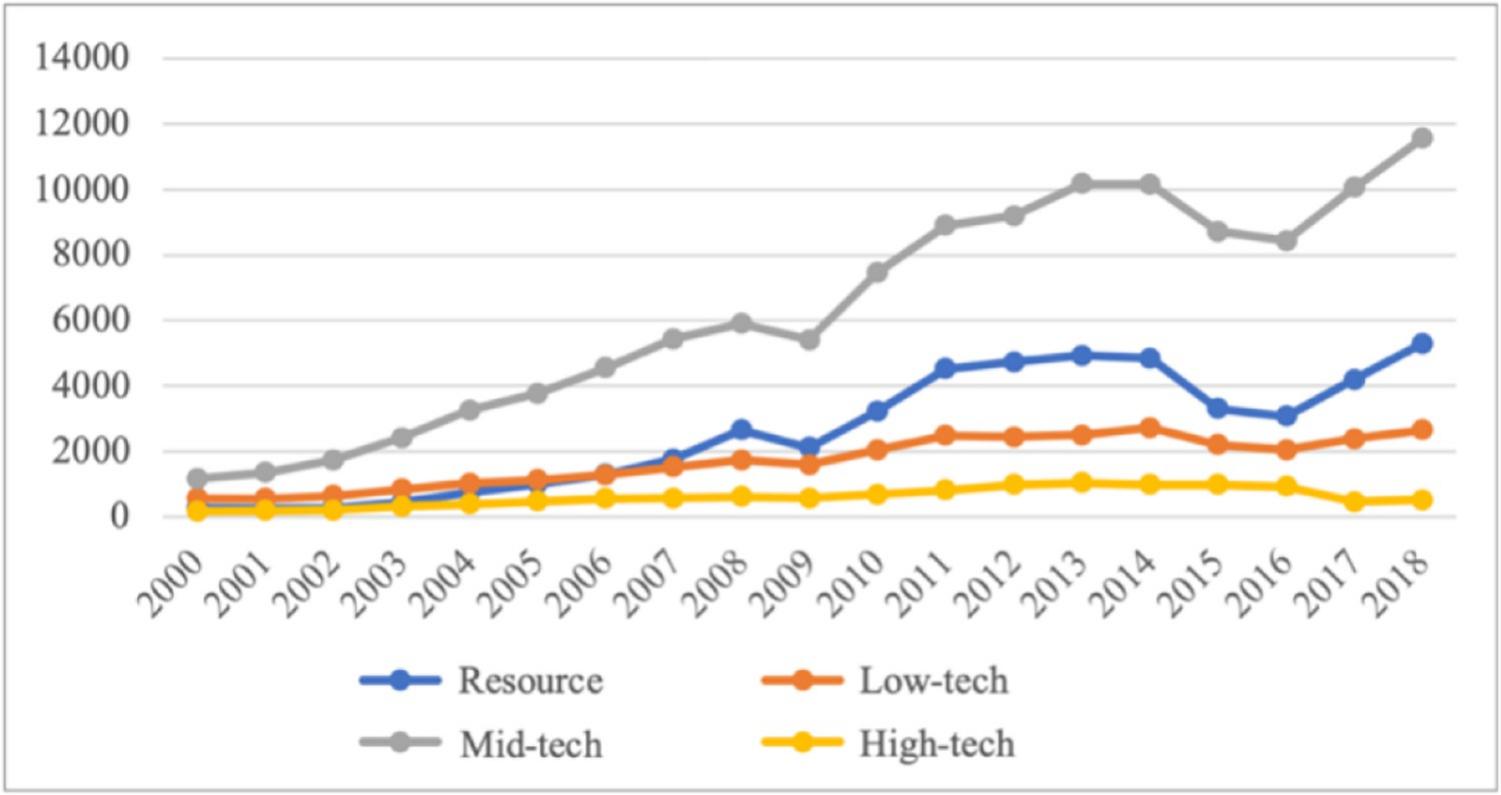
Analysis of the import trade structure of intermediate products in China’s industrial sectors from 2000 to 2018. Source: data were obtained from the WITS database.

Fig 3 reflects the trend of import trade value of intermediate reclassified products from 2000 to 2018. The four lines from top to bottom represent the import trade trend lines of medium-tech industrial products, resource products, low-tech industrial products and high-tech industrial products. It is evident that the trade trends of mid-tech industrial products, resource products and low-tech industrial products are almost the same. Before 2008, the import value of various products was rising at a relatively stable growth rate. The import value decreased from 2008 to 2009 and began to increase after 2009. Until 2014, the trade value fell due to the impact of prices. Since 2016, the trade value has continued to rise. And high-tech industrial products have not changed much in the past decade.

Moreover, in Fig 3, starting from 2000, mid-tech industrial manufactured products have been the most imported among intermediate products. Before 2004, the import scale of low-tech industrial products was higher than that of resource products, but after 2004, the import scale of resource products has always been second only to the import scale of mid-tech industrial products. With the development of the national economy, domestic producers have more and more demand for resources. When domestic resources cannot meet the demand, domestic producers will import foreign resource products, which may be the reason why the scale of imported resource products has been growing and exceeds the import scale of low technology industrial products.

### The sources of intermediate product imports

**Fig 4:**
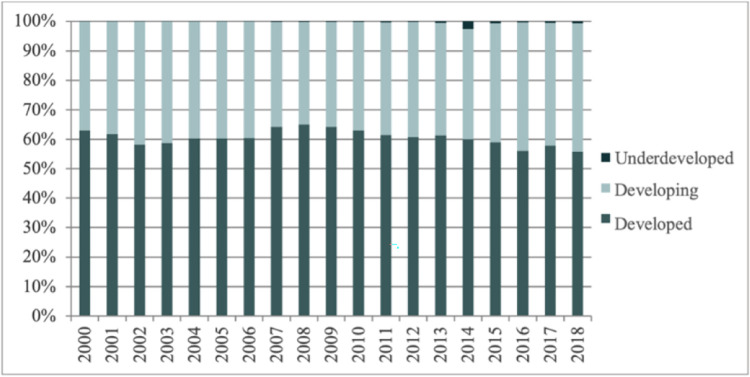
Proportion of import trade sources of intermediate products in the industrial sector from 2000 to 2018. Source: data were obtained from the WITS database.

Depending on different levels of economic development, the source countries of China’s intermediate product imports are classified as underdeveloped countries, developing countries and developed countries. As shown in Fig 4, the main source countries of China’s imports of intermediate goods are developed countries, and the proportion of intermediate products imported from developed countries has always been the largest and fluctuated around 60%, followed by developing countries, accounting for around 40%. Besides, the least proportion is the underdeveloped countries. Most of the top ten import source countries of industrial intermediate goods are developed countries.

**Fig 5:**
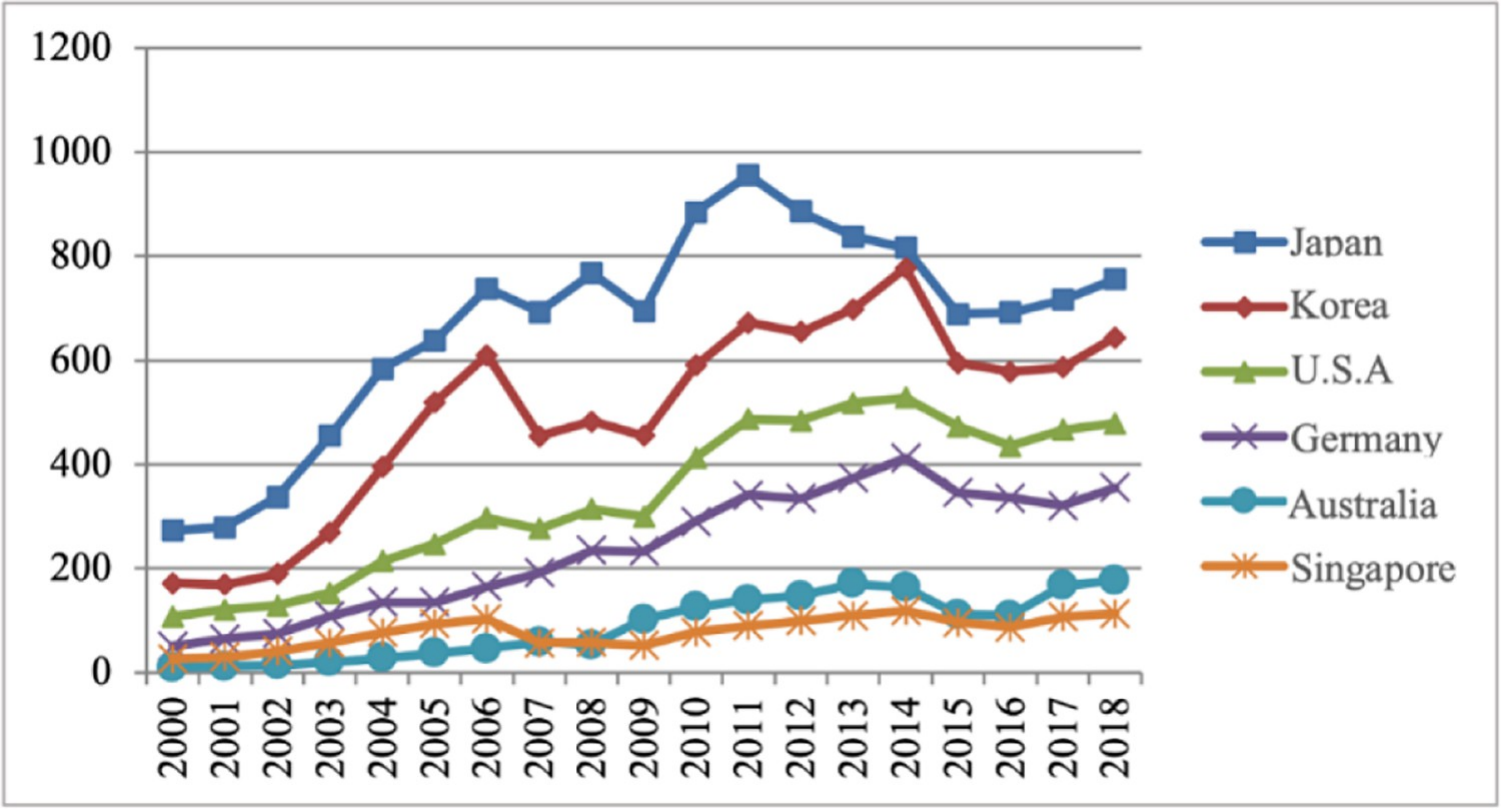
The total imports of intermediate products between my country and other countries from 2000 to 2018. Source: data were obtained from the WITS database.

Fig 5 shows the import trade volume of intermediate products between China’s industrial sectors and developed countries from 2000 to 2018. The developed countries in Fig 5 include Japan, South Korea, the United States, Germany, Australia, and Singapore. These developed countries are also the top 10 import source countries in terms of trade value of intermediate products in the industrial sectors. It can be seen that the overall import of intermediate products in the industrial sectors of my country and these developed countries has shown a growth trend. Among them, from 2000 to 2018, Japan has been the first source of imports of intermediate products in China’s industrial sectors, followed by South Korea, the United States, and Germany. Before 2007, the trade value of intermediate products imported by the industrial sectors from Singapore was more than that of Australia, but Australia surpassed Singapore in 2007.

## Empirical results

### GMM regression results

#### Regression results and analysis at the full-sample level

Based on the dynamic panel model, relying on the collected data on the import volume of intermediate goods and industrial pollution in 30 industrial sectors in China from 2003 to 2015, the emissions of industrial wastewater, industrial waste gas and industrial solid waste were regressed and estimated from the whole industry level through the systematic GMM method.

The results correspond to model (1), model (2) and model (3) in Table 3, respectively. It is not difficult to find through observation that intermediate product imports can effectively promote the emission reduction of three wastes. From models (1), (2) and (3), the impact of the core independent variable, import scale of intermediate goods on the emissions of three wastes in the industrial sectors, is significantly negative.

**Table 3:** Regression results of the impact of intermediate product imports on pollution emissions in the industrial sectors.

Variables	Model(1)		Model(2)		Model(3)	
	*lnFS*_*i*,*t*_		*lnFQ*_*i*,*t*_		*lnFG*_*i*,*t*_	
	Coefficient	z value	Coefficients	z value	Coefficients	z value
*lnFS*_*i*,*t*−1_	1.0879 ***	298.31				
	(0.0036)					
*lnFQ*_*i*,*t*−1_			1.0056 ***	307.84		
			(0.0032)			
*lnFG*_*i*,*t*−1_					1.0441***	392.46
					(0.0094)	
*lnIMIN*_*i*,*t*_	-0.0015***	-10.38	-0.0006**	-1.68	-0.0011***	-4.34
	(0.0001)		(0.0004)		(0.0003)	
*FDI*_*i*,*t*_	0.4214***	36.79	-0.2031***	-8.42	0.3794***	12.34
	(0.0115)		(0.0241)		(0.0307)	
ln*SIZE*_*i*,*t*_	-0.1140***	-19.09	0.0233***	2.80	-0.0263***	-6.26
	(0.0597)		(0.0083)		(0.0042)	
*lnFSERS*_*i*,*t*_	-0.0006	-0.14				
	(0.0044)					
*lnFQERS*_*i*,*t*_			-0.0399***	-5.70		
			(0.0070)			
*lnFGERS*_*i*,*t*_					-0.0840***	-13.20
					(0.0064)	
*lnRD*_*i*,*t*_	-0.0140***	-7.14	-0.0059***	-1.84	-0.0056***	-3.74
	(0.0020)		(0.0032)		(0.0150)	
*lnGDP*_*t*_	-5.2561***	-35.57	1.5998***	10.88	2.0455***	5.20
	(0.1478)		(0.1471)		(0.3932)	
*ln* ^2^ *GDP*_*t*_	0.2704	38.31	-0.1732***	-11.01	-0.1062***	-5.49
	(0.0071)		(0.0157)		(0.0194)	
*CONSTANT*	25.3596 ***	33.49	167.3466 ***	9.71	-8.7744 ***	-4.36
	(0.7572)		(17.2422)		(2.0120)	

Note: The values in parentheses are the standard deviations of the estimated coefficients of each variable, ***, **, and * indicate that the variables are significant at the test levels of 1%, 5%, and 10%, respectively.

In terms of control variables, (1) Foreign direct investment (FDI) has a significant negative correlation with industrial waste gas (FQ) emissions, and has a significant positive correlation with industrial wastewater (FS) and industrial solid waste (FG) emissions. This can reflect the different directions of foreign direct investment in industrial investment. One is that foreign investors tend to transfer resource mining industries as investment objects to developing countries like China. These industries have high short-term returns but large emissions of industrial wastewater and industrial solid waste. Second, foreign investors can enter industrial sectors by providing capital or technical support to improve the technical level of production processes and eventually reduce the pollution emission of industrial waste gases. (2) The regression coefficient of industry scale (SIZE) with industrial wastewater and industrial solid waste emissions is significantly negative, while industrial waste gas emissions are significantly positive. (3) The effects of three wastes environmental regulations (FSERS, FQERS, FGERS) on the corresponding pollution emissions (FS, FQ, FG) are all negatively correlated, but only the regression coefficients for industrial waste gas and industrial solid waste are obviously. (4) R&D investment (RD) has obvious effect on promoting emissions reduction of three wastes pollution. (5) The relationship between the level of economic development (GDP) and the emission of industrial wastewater, industrial waste gas and industrial solid waste presents a positive U type, an inverted U type and an inverted U type respectively. As can be seen from Table 3, the regression coefficients of the primary and quadratic terms of all economic development levels are significant. In model (1), the level of economic development and industrial wastewater emission are in a positive U shape, which is inconsistent with the traditional environmental Kuznets curve theory. This may be because it is still on the left side of the inverted U shape, not passing the inflection point of the curve, and is in the rising stage. The level of economic development and industrial waste gas and industrial solid waste shows an inverted U shaped curve, which is in line with the traditional environmental Kuznets curve law, and is now on the right side of U. With the increase of the level of economic development, the emissions of industrial waste gas and industrial solid waste will decrease.

### Regression results and analysis at the sub-sample level

For industrial sectors with different pollution levels, intermediate product imports will also have heterogenous effects on them. Refer to the practice of Wang [36], the 30 industries are divided into heavily, moderately, lightly polluted industries by the intensity of pollution emissions. The regression analysis is conducted on these three subsamples to explore the relationship between intermediate product imports and industrial pollution emissions with different pollution levels.

The estimated results are shown in Tables 4–6. The regression coefficients of intermediate product imports in the heavy pollution industries to pollution emissions are all significantly negative at the level of 1%, which means that intermediate product imports can promote the reduction of industrial pollutants in the heavily polluted industries. The elimination of industrial solid waste emission is the most visible of them. Heavily polluted industries like coal mining, nonferrous metal mining, ferrous metal mining, and smelting generate more industrial solid wastes than other industries. However, the import of intermediate products lowers the mining industry’s mining volume, which to some extent lowers the industrial solid waste emissions. In the estimation of the moderately polluted industries, the impact of intermediate product imports on the emission reduction of the "three wastes" is ranked in descending order, namely industrial waste gas, industrial solid waste, and industrial wastewater. While lightly polluting industries are industrial wastewater, industrial waste gas, and industrial solid waste.

**Table 4:** Regression results of the impact of intermediate product imports on pollution emissions in the heavily polluted industries.

Variable	Model (4)		Model (5)		Model (6)	
	*lnFS*_*i*,*t*_(4)		*lnFQ*_*i*,*t*_		*lnFG*_*i*,*t*_	
	coefficients	z value	coefficients	z value	coefficients	z value
*lnFS*_*i*,*t*−1_	1.1287 ***	304.29				
	(0.0037)					
*lnFQ*_*i*,*t*−1_			1.0562***	123.34		
			(0.0086)			
*lnFG*_*i*,*t*−1_					1.0444***	464.00
					(0.0023)	
*high* ×*lnIMIN*_*i*,*t*_	-0.0165***	-35.49	-0.0037***	-2.79	-0.0041***	-6.87
	(0.0005)		(0.0013)		(0.0010)	
*FDI*_*i*,*t*_	0.2435***	38.04	-0.0662**	-2.26	0.3108***	18.04
	(0.0064)		(0.0256)		(0.0172)	
*SIZE*_*i*,*t*_	-0.1791***	-30.01	0.0677***	5.73	-0.0252***	-7.60
	(0.0060)		(0.0609)		(0.0033)	
*lnFSER*_*i*,*t*_	-0.0465***	-11.33				
	(0.0041)					
*lnFQER*_*i*,*t*_			-0.0005	-0.14		
			(0.0040)			
*lFGER*_*i*,*t*_					-0.0850***	-16.31
					(0.0052)	
*lnRD*_*i*,*t*_	-0.1135***	-19.05	-0.0436***	-8.49	-0.0056***	-4.15
	(0.0060)		(0.0051)		(0.0013)	
*lnGDP*_*t*_	-0.3196***	-20.12	11.6852***	12.15	2.5252***	6.67
	(0.0159)		(0.9619)		(0.3786)	
*ln* ^2^ *GDP*_*t*_	0.0363***	25.52	-0.5666***	-12.15	-0.1303***	-7.06
	(0.0014)		(0.0466)		(0.0185)	
*CONSTANT*	0.7998 ***	11.48	2.5345 ***	17.68	-11.1448 ***	-5.69
	(0.0697)		(0.1433)		(1.9574)	

Note: The values in parentheses are the standard errors of the estimated coefficients of each variable, ***, **, and * indicate that the variables are significant at the test levels of 1%, 5%, and 10%, respectively.

**Table 5:** Regression results of the impact of intermediate product imports on pollution emissions in the moderately polluted industries.

variable	Model (7)		Models (8)		Models (9)	
	*lnFS*_*i*,*t*_		*lnFQ*_*i*,*t*_		*lnFG*_*i*,*t*_	
	coefficients	z value	coefficients	z value	coefficients	z value
*lnFS*_*i*,*t*−1_	1.0213 ***	299.82				
	(0.0034)					
*lnFQ*_*i*,*t*−1_			0.8866 ***	26.87		
			(0.0330)			
*lnFG*_*i*,*t*−1_					0.9233 ***	244.90
					(0.0038)	
*med* ×*lnIMIN*_*i*,*t*_	-0.0007***	-3.08	-0.0088***	-10.80	-0.0071***	-23.12
	(0.0002)		(0.0008)		(0.0003)	
*FDI*_*i*,*t*_	0.2475***	23.89	-0.8822***	-4.49	-1.0376***	-22.59
	(0.0002)		(0.1966)		(0.0459)	
*SIZE*_*i*,*t*_	0.0028***	0.55	0.1957***	4.16	0.0198***	-5.19
	(0.0050)		(0.0471)		(0.0038)	
*lFSER*_*i*,*t*_	-0.0933	-23.15				
	(0.0040)					
*lnFQER*_*i*,*t*_			-0.0068	-1.45		
			(0.0047)			
*lnFGER*_*i*,*t*_					-0.1111***	-18.52
					(0.0060)	
*lnRD*_*i*,*t*_	-0.0255***	-16.88	-0.0185***	-4.75	-0.0737***	-25.70
	(0.0015)		(0.0039)		(0.0029)	
*lnGDP*_*t*_	-12.3003	-52.35	-14.5772***	-6.99	11.4747***	22.76
	(0.2541)		(2.0865)		(0.5042)	
*ln* ^2^ *GDP*_*t*_	0.6455***	53.86	0.6753***	6.43	-0.5990***	-23.89
	(0.0120)		(0.1051)		(0.0251)	
*CONSTANT*	67.2033***	51.10	78.1782***	7.46	-53.2513 ***	-21.02
	(1.3152)		(10.4790)		(2.5336)	

Note: The values in parentheses are the standard errors of the estimated coefficients of each variable, ***, **, and * indicate that the variables are significant at the test levels of 1%, 5%, and 10%, respectively.

**Table 6:** Regression results of the impact of intermediate product imports on pollution emissions in the lightly polluted industries.

variable	Models (10)		Models (11)		Models (12)	
	*lnFS*_*i*,*t*_		*lnFQ*_*i*,*t*_		*lnFG*_*i*,*t*_	
	coefficients	z value	coefficients	z value	coefficients	z value
*lnFS*_*i*,*t*−1_	0.9826 ***	855.18				
	(0.0011)					
*lnFQ*_*i*,*t*−1_			0.9786 ***	164.61		
			(0.0059)			
*lnFG*_*i*,*t*−1_					1.0256 ***	573.44
					(0.0018)	
*low* ×*lnIMIN*_*i*,*t*_	-0.0021 ***	-9.52	-0.0019 **	-2.31	-0.0010 **	-2.04
	(0.0002)		(0.0008)		(0.0005)	
*FDI*_*i*,*t*_	0.2003***	54.55	-0.2951***	-10.98	0.1930***	10.97
	(0.0037)		(0.0269)		(0.0176)	
*SIZE*_*i*,*t*_	0.0809***	36.80	0.0329***	4.70	-0.0021	-0.93
	(0.0022)		(0.0070)		(0.0023)	
*lnFSER*_*i*,*t*_	0.2438	53.35				
	(0.0046)					
*lnFQER*_*i*,*t*_			-0.0028***	-0.70		
			(0.0039)			
*lnFGER*_*i*,*t*_					-0.0792***	-15.35
					(0.0052)	
*lnRD*_*i*,*t*_	-0.0505***	-49.05	-0.0264***	-9.88	-0.0106***	-8.09
	(0.0010)		(0.0027)		(0.0013)	
*lnGDP*_*t*_	-32.3591***	-64.14	-11.1304***	-11.51	3.7635***	9.46
	(0.5045)		(0.9671)		(0.3978)	
*ln* ^2^ *GDP*_*t*_	1.5610***	64.15	0.5286	11.30	-0.1932***	-10.00
	(0.0243)		(0.0468)		(0.0193)	
*CONSTANT*	33.1569***	65.63	58.4427***	11.64	17.2983***	-8.39
	(0.5052)		(5.0228)		(2.0622)	

Note: The values in parentheses are the standard errors of the estimated coefficients of each variable, ***, **, and * indicate that the variables are significant at the test levels of 1%, 5%, and 10%, respectively.

For control variables, (1) The entry of foreign direct investment (FDI) promotes the emission reduction of industrial waste gas in heavily polluted industries, but has a significant effect on increasing the emission of industrial wastewater and industrial solid waste. It can increase the emission of industrial wastewater from moderately polluted industries, but can reduce the emission of other two types of wastes. It aggravates the emission of industrial wastewater and industrial solid waste in lightly polluted industries, and reduces the emission of industrial waste gas. (2) The regression coefficients of industry scale (SIZE) on industrial wastewater and industrial solid waste emissions in heavily polluted industries are significantly negative, and the relationship with industrial waste gas emissions is positively correlated. And it can significantly increase the emission of three wastes in moderately polluted industries. There is a significant positive correlation between industrial wastewater and industrial exhaust emissions in light pollution industries, while there is no significant impact on industrial solid waste emissions. (3) Under environmental regulations (FGERS, FSERS, FQERS), the treatment of industrial wastewater and industrial solid waste in heavily polluted industries is effective, and it can absorb experience while dealing with the current generation to reduce the next industrial wastewater in the next phase. However, the industry does not pay attention to the treatment of industrial exhaust emissions, and because it is "pollution first, treatment later", thus failing to promote emission reduction industrial solid waste in moderately polluted industries The regression coefficient of environmental regulation (FGERS) is negative at the 1% level, while industrial wastewater environmental regulation (FSERS) and industrial waste gas environmental regulation (FQERS) have no significant effect, but the regression coefficients are all negative. It is obvious that the treatment effect of industrial solid waste in moderately polluted industries is effective; the emission of industrial waste gas and industrial solid waste has been effectively treated in lightly polluted industries. (4) The increase in R&D investment (RD) has a significant role in promoting the emissions reduction of three wastes in the three varieties of polluting industries. (5) The level of economic development (GDP) with the emission of industrial wastewater, industrial waste gas and industrial solid waste in heavily and lightly polluted industries show a positive U shape, an inverted U shape and an inverted U shape, respectively. This is also consistent with the results of economic development level and three wastes emissions in the above analysis.

### Robustness test

We conducted multiple robustness tests on the regression results of each sample by changing the sample inspection period. Adjusting the research period from 2003–2015 to 2005–2015, using the same regression method, Tables 7 and 8 were obtained.

**Table 7:** Robustness test of research on the impact of intermediate product imports on pollution emissions from heavy and moderately polluting industries.

variable	heavily polluted industries			moderately polluted industries		
	Model (13)	Model (14)	Model (15)	Model (16)	Model (17)	Model (18)
	*lnFS*_*i*,*t*_	*lnFQ*_*i*,*t*_	*lnFG*_*i*,*t*_	*lnFS*_*i*,*t*_	*lnFQ*_*i*,*t*_	*lnFG*_*i*,*t*_
*lnFS*_*i*,*t*−1_	1.0511***			1.0337***		
	(0.0019)			(0.0023)		
*lnFQ*_*i*,*t*−1_		1.0103***			0.9782***	
		(0.0058)			(0.0003)	
*lnFG*_*i*,*t*−1_			1.0343***			0.8857***
			(0.0048)			(0.0032)
*high* ×*lnIMIN*_*i*,*t*_	-0.0078***	-0.0021***	-0.0014**			
	(0.0002)	(0.0009)	(0.0013)			
*med* ×*lnIMIN*_*i*,*t*_				-0.0011***	-0.0013***	-0.0092***
				(0.0002)	(0.0003)	(0.0003)
*FDI*_*i*,*t*_	0.1838***	-0.0044***	0.2076***	0.3115***	-0.1216***	-1.5659***
	(0.0045)	(0.0232)	(0.0360)	(0.0070)	(0.0106)	(0.0414)
*SIZE*_*i*,*t*_	-0.0776***	0.0498***	-0.0074	0.0374***	0.0541***	-0.93
	(0.0031)	(0.0083)	(0.0079)	(0.0034)	(0.0056)	0.2623***
*lnFSER*_*i*,*t*_	-0.2438***			0.1594***		(0.0072)
	(0.0046)			(0.0046)		
*lnFQER*_*i*,*t*_		-0.0016			-0.0180***	
		(0.0035)			(0.0035)	
*lnFGER*_*i*,*t*_			-0.1256***			-0.1297***
			(0.0056)			(0.0062)
*lnRD*_*i*,*t*_	-0.0249***	-0.0271***	-0.0118***	-0.0046***	-0.0064**	-0.0935***
	(0.0013)	(0.0020)	(0.0025)	(0.0012)	(0.0026)	(0.0030)
*lnGDP*_*t*_	-2.5008***	4.1821***	8.6620***	-0.7117***	-25.0089***	5.8925***
	(0.0878)	(0.7212)	(0.9671)	(0.0167)	(2.0784)	(0.9044)
*ln* ^2^ *GDP*_*t*_	0.1351***	0,2059***	-0.4295***	0.0477***	-1.2011***	-0.3434***
	(0.0043)	(0.0346)	(0.0468)	(0.0012)	(0.1000)	(0.0431)
*CONSTANT*	9.7748***	21.3791***	-42.1090***	13.9545***	130.2628***	-22.7002***
	(0.4661)	(3.7601)	(2.4088)	(0.5573)	(10.8023)	(4.7473)

Note: The values in parentheses are the standard errors of the estimated coefficients of each variable, ***, **, and * indicate that the variables are significant at the test levels of 1%, 5%, and 10%, respectively.

**Table 8:** Robustness test of research on the impact of intermediate product imports on pollution emissions from lightly polluting industries.

variable	lightly polluting industries		
	Model (19)	Model (20)	Model (21)
	*lnFS*_*i*,*t*_	*lnFQ*_*i*,*t*_	*lnFG*_*i*,*t*_
*lnFS*_*i*,*t*−1_	0.9945***		
	(0.0011)		
*lnFQ*_*i*,*t*−1_		0.9763***	
		(0.0042)	
*lnFG*_*i*,*t*−1_			0.9831***
			(0.0017)
*low* ×*lnIMIN*_*i*,*t*_	-0.0004*	-0.0045***	-0.0101**
	(0.0002)	(0.0006)	(0.0005)
*FDI*_*i*,*t*_	0.2058***	-0.0892***	0.1248***
	(0.0039)	(0.0191)	(0.0190)
*SIZE*_*i*,*t*_	0.0296***	0.0167***	-0.0472***
	(0.0017)	(0.0052)	(0.0027)
*lnFSER*_*i*,*t*_	0.1460***		
	(0.0044)		
*lnFQER*_*i*,*t*_		-0.0075**	
		(0.0035)	
*lnFGER*_*i*,*t*_			-0.0595***
			(0.0053)
*lnRD*_*i*,*t*_	-0.2045***	-0.0373***	-0.0085***
	(0.0009)	(0.0027)	(0.0014)
*lnGDP*_*t*_	-11.2113***	-4.7708***	0.5652***
	(0.2931)	(0.5729)	(0.0195)
*ln* ^2^ *GDP*_*t*_	0.5431***	0.2317***	-0.0488***
	(0.0140)	(0.0276)	(0.0014)
*CONSTANT*	55.8168***	24.6323***	-16.3418***
	(1.5487)	(2.9795)	(0.8879)

Note: The values in parentheses are the standard errors of the estimated coefficients of each variable, ***, **, and * indicate that the variables are significant at the test levels of 1%, 5%, and 10%, respectively.

### Mechanism test

In the theoretical analysis of the mechanism in Methodology, intermediate product imports impact on the pollution emissions of the industrial sectors through the competition effect, variety effect and technology spillover effect. Since the competition effect can be directly measured by the import trade volume, only the variety index of intermediate product imports and the technology spillover index of intermediate product imports are constructed to test. Finally, the three types of import effects are included in the dynamic mediation effect model as mediating variables to explore the indirect impact channels of intermediate product imports on industrial pollution emissions.

### Variety effect

Table 9 shows the regression results of indirect impact of intermediate product imports on industrial pollution charges based on the diversity of imports. Model (16), model (17) and model (18) are the results of the dynamic mediation model based on the panel data of industrial wastewater, waste gas, and solid waste, respectively. Among them, the results in (1), (3) and (5) are the impact models of intermediate product imports on the diversity of imported intermediates, and (2), (4) and (6) are the regression models after adding the mediating variable, import variety effect index.

**Table 9:** Regression results of the mediation effect test based on the variety effect.

variable	Model (22)		Model (23)		Model (24)	
	(1)	(2)	(3)	(4)	(5)	(6)
	*DI*_*i*,*t*_	*lnFS*_*i*,*t*_	*DI*_*i*,*t*_	*lnFQ*_*i*,*t*_	*DI*_*i*,*t*_	*lnFG*_*i*,*t*_
*lnFS*_*i*,*t*−1_		1.0290 ***				
		(0.0035)				
*lnFQ*_*i*,*t*−1_				1.0173 ***		
				(0.0017)		
*lnFG*_*i*,*t*−1_						1.0671***
						(0.0060)
*DI*_*i*,*t*_		-0.0173***		-0.0189***		-0.0163***
		(0.0012)		(0.0023)		(0.0015)
*DI*_*i*,*t*−1_	1.0804***		1.1734***		1.1490***	
	(0.0069)		(0.0324)		(0.0182)	
*lnIMIN*_*i*,*t*_	0.0005***	-0.0008***	0.0011***	-0.0007*	0.0004***	-0.0019***
	(0.0001)	(0.0001)	(0.0002)	(0.0004)	(0.0001)	(0.0003)
*FDI*_*i*,*t*_	0.0382***	0.2604***	0.0100**	-0.0941***	0.0095***	0.6488***
	(0.0035)	(0.0109)	(0.0042)	(0.0153)	(0.0035)	(0.0698)
*lnSIZE*_*i*,*t*_	0.0793***	-0.0090***	0.1166	0.3211***	0.0734***	-0.0562***
	(0.0052)	(0.0053)	(0.0982)	(0.0567)	(0.0094)	(0.0105)
*lnFSERS*_*i*,*t*_	0.0532***	-0.0241*				
	(0.0017)	(0.0036)				
*lnFQERS*_*i*,*t*_			0.0135***	-0.0101**		
			(0.0031)	(0.0040)		
*lnFGERS*_*i*,*t*_					0.0705***	-0.0798***
					(0.0011)	(0.0061)
*lnRD*_*i*,*t*_	-0.0043***	-0.1562***	-0.0522***	-0.0140***	-0.0052***	-0.0052
	(0.0007)	(0.0193)	(0.0097)	(0.0024)	(0.0007)	(0.0032)
*lnGDP*_*t*_	0.4172***	-8.7615***	-0.8683***	15.1532***	0.5245***	0.2211***
	(0.0138)	(0.1477)	(0.1912)	(1.4273)	(0.1428)	(0.0462)
*ln* ^2^ *GDP*_*t*_	-0.0314***	0.4246***	0.0363***	-0.7573***	-0.0235***	-0.0121***
	(0.0012)	(0.0072)	(0.0096)	(0.0688)	(0.0064)	(0.0040)
*Constant*	0.6455 ***	0.6502 ***	1.7288	76.2941 ***	-2.1961 ***	-23.2198 ***
	(0.0813)	(0.1447)	(9.5326)	(7.4384)	(0.8156)	(2.1835)

Note: The values in parentheses are the standard errors of the estimated coefficients of each variable, ***, **, and * indicate that the variables are significant at the test levels of 1%, 5%, and 10%, respectively.

Observing the mediating variable and core independent variable in the table, we found that with the data of different pollutants, the regression coefficients of the import variety effect index (DI_it_) for one lag period are all significantly positive, and intermediate product imports have a significant effect on the import species. With the expansion of the import scale of intermediate products, the more varieties and quantities of imports brought about by intermediate product imports, the greater the index of import variety effect will be. In the overall regression model with the mediating variables, that is, in the results of columns (2), (4), and (6), the regression coefficients of intermediate product imports on industrial three wastes pollution emissions are still significantly negative. Furthermore, the increase of import category can reduce the emissions of three wastes pollutants in the industrial sectors, indicating that there is a significant partial mediating role.

### Technology spillovers effect

Using the technical spillover effect index of imported intermediate products as the mediating variable, the dynamic mediation effect model is regressed. Table 10 reports the regression results. The regression coefficients of the import technology spillover effect index (TEC_it_) for one lag period are all significantly positive. Relying on the panel data of different pollutants, the relationship between intermediate product imports and the import technology spillover effect index is positively correlated. After adding the mediating variable, intermediate product imports still play a role in promoting the emissions reduction of three wastes pollution. In addition, the improvement of imported technology spillover index can reduce the emissions of three wastes in the industrial sectors. The regression coefficients of intermediate product imports and technology spillover indicators are both significantly negative, so the technology spillover effect plays a partial intermediary role in the industrial three wastes emissions.

**Table 10:** Regression results of the mediation effect test based on technology spillovers effect.

variable	Model (25)		Model (26)		Model (27)	
	(1)	(2)	(3)	(4)	(5)	(6)
	*TEC*_*i*,*t*_	*lnFS*_*i*,*t*_	*TEC*_*i*,*t*_	*lnFQ*_*i*,*t*_	*TEC*_*i*,*t*_	*lnFG*_*i*,*t*_
*lnFS*_*i*,*t*_		0.0000 ***				
		(0.0000)				
*lnFQ*_*i*,*t*_				0.7037 ***		
				(0.0315)		
*lnFG*_*i*,*t*_						1.0052***
						(0.0008)
*TEC*_*i*,*t*_		-0.0483***		-0.0229***		-0.0152***
		(0.0019)		(0.0019)		(0.0004)
*TEC*_*i*,*t*−1_	1.0379***		0.6229***		0.6919***	
	(0.0058)		(0.0444)		(0.0440)	
*lnIMIN*_*i*,*t*_	0.0020**	-0.0026***	0.0207***	-0.0007***	0.0170***	-0.0008***
	(0.0009)	(0.0006)	(0.0024)	(0.0004)	(0.0024)	(0.0002)
*FDI*_*i*,*t*_	0.2300***	0.3549*	1.2512***	-2.0530***	1.0196***	0.1237***
	(0.0243)	(0.0493)	(0.1600)	(0.1941)	(0.1591)	(0.0131)
*lnSIZE*_*i*,*t*_	0.1832***	-0.0006	-0.2025***	0.4395***	-0.1457***	-0.0504***
	(0.0066)	(0.0295)	(0.0391)	(0.0455)	(0.0390)	(0.0025)
*lnFSERS*_*i*,*t*_	0.3898***	-0.0006				
	(0.0088)	(0.0216)				
*lnFQERS*_*i*,*t*_			0.1155***	0.0029		
			(0.0030)	(0.0045)		
*lnFGERS*_*i*,*t*_					0.2548***	-0.1068***
					(0.0224)	(0.0053)
*lnRD*_*i*,*t*_	-0.1360***	-0.0334***	-0.2636***	-0.0227***	-0.2032***	-0.0366***
	(0.0060)	(0.0100)	(0.0414)	(0.0043)	(0.0410)	(0.0014)
*lnGDP*_*t*_	13.7561***	-14.5259***	20.5326***	5.3759***	21.7952***	3.9828***
	(0.6594)	(1.0282)	(0.4396)	(1.3148)	(0.9615)	(0.3217)
*ln* ^2^ *GDP*_*t*_	-0.6855***	0.7138***	-0.8979***	-0.1947***	-0.9877***	-0.2161***
	(0.0304)	(0.0486)	(0.0792)	(0.0673)	(0.0532)	(0.0156)
*Cnstant*	-70.6582 ***	84.1796 ***	-110.6173 ***	33.3336 ***	-119.6069 ***	-16.9674 ***
	(3.3629)	(5.2025)	(6.9474)	(6.5300)	(4.7609)	(1.6775)

Note: The values in parentheses are the standard errors of the estimated coefficients of each variable, ***, **, and * indicate that the variables are significant at the test levels of 1%, 5%, and 10%, respectively.

### Robustness test

Due to the possibility of some outliers in the original data, resulting in differences between the observed data and the actual data, the original sample data is subjected to a "tail reduction" approach, replacing<1% of the values in the sample data with their 1-percentile values and>99% with values in their 99th percentile [37] to test the robustness of the mediation effect model.

Table 11 shows the robustness test of the regression results of the intermediary model based on the variety effect indicator of intermediate goods imports.

**Table 11:** Robustness test of the regression results of the intermediary model based on the effect index of the imported intermediate products.

variable	Model(28)		Model(29)		Model(30)	
	(1)	(2)	(3)	(4)	(5)	(6)
	*DI*_*i*,*t*_	*lnFS*_*i*,*t*_	*DI*_*i*,*t*_	*lnFQ*_*i*,*t*_	*DI*_*i*,*t*_	*lnFG*_*i*,*t*_
*lnFS*_*i*,*t*−1_		1.0646***				
		(0.0035)				
*lnFQ*_*i*,*t*−1_				1.0385***		
				(0.0031)		
*lnFG*_*i*,*t*−1_						1.0603***
						(0.0095)
*DI*_*i*,*t*_		-0.0293***		-0.0328***		-0.0170***
		(0.0013)		(0.0028)		(0.0035)
*DI*_*i*,*t*−1_	0.8741***		1.0124***		1.1078***	
	(0.0044)		(0.0058)		(0.0198)	
*lnIMIN*_*i*,*t*_	0.0003***	-0.0012***	0.0001*	-0.0014***	0.0002***	-0.0018***
	(0.0001)	(0.0001)	(0.0000)	(0.0005)	(0.0001)	(0.0004)
*FDI*_*i*,*t*_	0.1046***	0.3748***	0.0188**	-0.2309***	0.0340***	0.5699***
	(0.0040)	(0.0130)	(0.0025)	(0.0208)	(0.0119)	(0.1117)
*lnSIZE*_*i*,*t*_	0.0720***	-0.0529***	0.0414***	0.0729***	0.0634***	-0.0372***
	(0.0027)	(0.0058)	(0.0982)	(0.0402)	(0.0121)	(0.0135)
*lnFSERS*_*i*,*t*_	0.0462***	-0.0334*				
	(0.0013)	(0.0046)				
*lnFQERS*_*i*,*t*_			0.0060***	-0.1205**		
			(0.0005)	(0.0282)		
*lnFGERS*_*i*,*t*_					0.0716***	-0.0447***
					(0.0009)	(0.0051)
*lnRD*_*i*,*t*_	-0.0013***	-0.0040***	-0.0042**	-0.0010	-0.0009**	-0.0041
	(0.0004)	(0.0023)	(0.0018)	(0.0019)	(0.0004)	(0.0043)
*lnGDP*_*t*_	0.3601***	-7.7044***	-0.6200***	33.0875***	1.4229***	2.1931***
	(0.0066)	(0.2145)	(0.0386)	(3.9420)	(0.2994)	(0.7522)
*ln* ^2^ *GDP*_*t*_	-0.0280***	0.3823***	0.0278***	-1.6119***	-0.0679***	-0.1094***
	(0.0006)	(0.0101)	(0.0019)	(0.1915)	(0.0135)	(0.0385)
*Constant*	12.2558***	38.3096***	3.5565***	-168.2235***	-6.6069***	-10.3942***
	(0.2927)	(1.1064)	(0.2022)	(20.1818)	(1.6667)	(3.6507)

Note: The values in parentheses are the standard errors of the estimated coefficients of each variable, ***, **, and * indicate that the variables are significant at the test levels of 1%, 5%, and 10%, respectively.

Table 12 shows the robustness test of the regression results of the intermediary model based on the intermediate import technology spillover effect indicator. The conclusions obtained are consistent with those obtained from the mediation regression results in the previous section, indicating that the conclusions in the previous section are reliable and effective.

**Table 12:** Robustness test of the regression results of the intermediary model based on the technology spillover effect index of imported intermediate products.

variable	Model (31)		Model (32)		Model (33)	
	(1)	(2)	(3)	(4)	(5)	(6)
	*TEC*_*i*,*t*_	*lnFS*_*i*,*t*_	*TEC*_*i*,*t*_	*lnFQ*_*i*,*t*_	*TEC*_*i*,*t*_	*lnFG*_*i*,*t*_
*lnFS*_*i*,*t*_		1.1737***				
		(0.0057)				
*lnFQ*_*i*,*t*_				0.9261***		
				(0.0233)		
*lnFG*_*i*,*t*_						1.0270***
						(0.0080)
*TEC*_*i*,*t*_		-0.0072***		-0.0170***		-0.0114***
		(0.0004)		(0.0014)		(0.0018)
*TEC*_*i*,*t*−1_	1.0499***		0.9345***		0.6063***	
	(0.0043)		(0.0223)		(0.0485)	
*lnIMIN*_*i*,*t*_	0.0007**	-0.0026***	0.0040***	-0.0007**	0.0185***	-0.0012***
	(0.0003)	(0.0002)	(0.0009)	(0.0004)	(0.0022)	(0.0003)
*FDI*_*i*,*t*_	0.0673***	0.7294***	0.3151***	-0.7550***	1.6659***	0.1231
	(0.0216)	(0.0206)	(0.0832)	(0.1419)	(0.1944)	(0.1003)
*lnSIZE*_*i*,*t*_	0.2639***	-0.2736***	-0.0968***	0.1609***	-0.2821***	-0.0170***
	(0.0063)	(0.0105)	(0.0260)	(0.0370)	(0.0556)	(0.0152)
*lnFSERS*_*i*,*t*_	0.4787***	-0.0973***				
	(0.0080)	(0.0059)				
*lnFQERS*_*i*,*t*_			0.0321***	0.0029		
			(0.0069)	(0.0033)		
*lnFGERS*_*i*,*t*_					0.2978***	-0.0654***
					(0.0281)	(0.0051)
*lnRD*_*i*,*t*_	-0.1752***	-0.0173***	-0.0253***	-0.0370***	-0.3446***	-0.0310***
	(0.0054)	(0.0024)	(0.0069)	(0.0056)	(0.0543)	(0.0058)
*lnGDP*_*t*_	0.2287***	-0.5256***	32.6938***	5.6656***	34.3204***	4.1932***
	(0.0245)	(0.0256)	(0.6115)	(0.7779)	(1.3506)	(0.5757)
*ln* ^2^ *GDP*_*t*_	-0.0483***	0.0624***	-1.6060***	-0.2467***	-1.5537***	-0.2185***
	(0.0026)	(0.0026)	(0.0327)	(0.0400)	(0.0653)	(0.0302)
*Constant*	47.4229***	54.3099***	-165.3310***	31.7755***	-188.5901***	-19.2780***
	(3.5435)	(1.6866)	(3.0970)	(3.8620)	(7.4640)	(2.7267)

Note: The values in parentheses are the standard errors of the estimated coefficients of each variable, ***, **, and * indicate that the variables are significant at the test levels of 1%, 5%, and 10%, respectively.

Based on the above results, this paper confirms that the import trade of intermediate products can significantly reduce the pollution emission intensity of the three wastes in the industrial sectors. Through the inspection of the control variables, it is found that the R&D investment has an inhibitory effect on the three pollution emissions, indicating that no matter whether the enterprise improves the front-end treatment of the production process or installs the pollution control equipment for the end treatment, a large amount of investment is required. The difference in the impact direction of the three types of pollutants in foreign direct investment reflects that while foreign investors provide advanced clean technologies and production processes, they tend to choose resource mining industries as investment targets to transfer pollution to China, resulting in insignificant inhibition effects. Further, the different results obtained by regressing the sub-samples will help government departments to implement differentiated strategies according to industry heterogeneity, providing new ideas for accelerating the green and low-carbon transformation of manufacturing. Finally, the results of the mechanism test show that the liberalization of intermediate products trade promotes industrial pollution reduction through the competition effect, variety effect and technology spillover effect, which better verifies the theoretical mechanism of this paper, and has certain enlightenment significance for China to promote the reform of intermediate trade system and its own carbon peak in the industrial field.

## Conclusion and discussion

### Conclusion

This paper studies the impact and mechanism of intermediate product imports on industrial three wastes pollution charges through dynamic regression model and mediation effect model, and draws the following research conclusions:

First, it is found that the emission of three wastes in the industrial sectors is significantly reduced by intermediate product imports. More subtly, the R&D investment reduces the amount of all three pollutants emissions. Foreign direct investment can help cut down on industrial waste gas and solid waste emissions. While only the emission of industrial wastewater is negatively impacted by the size of the industry. The environmental regulation of industrial solid waste can effectively reduce industrial solid waste production, whereas the level of economic development affects the discharge of industrial wastewater, industrial waste gas, and industrial solid waste, exhibiting a positive U-shaped, inverted U-shaped, and inverted U-shaped curve.

Second, for heavily, moderately and lightly polluted industries with different levels of pollution, intermediate product imports have different negative impacts on three industrial pollution emissions. Among them, the import of intermediate products has a greater negative impact on the emission of industrial solid waste in the heavily polluted industries; In the medium and light pollution industries, the import of intermediate products has a greater impact on the reduction of industrial emissions. In the field of control variables, the impact of R&D investment on the emission of different pollutants in industrial sectors with different pollution levels is significantly negative, and the impact of other control variables on the emission of different pollutants in industrial sectors with different pollution levels is different.

Third, a mediation effect model is constructed to test the mechanism. The results show that the import trade of intermediate products reduces the emission intensity of three wastes in the industrial sectors through the competition effect, variety effect and technology spillover effect, which is consistent with the theoretical expectation.

### Discussion

Since intermediate product imports has significantly reduced the pollution emissions of China’s industrial sectors on the whole, continuing to expand intermediate product imports is an important policy measure for improving production conditions and reducing pollution at the source of production. In this regard, this paper proposes the following policy recommendations:

First, expand the import scale of intermediate products and increase the proportion of high-tech intermediate products. In order to deepen the reform of intermediate trade liberalization, we should continue to promote the reform and innovation of free trade pilot zones, optimize the structure while expanding the import scale of intermediate products, increase the import scale of high-tech products in industrial manufactured products, and obtain technology spillovers from them. First, we should reduce the import tariffs of high-tech intermediate products, reduce the import costs of enterprises, and increase the scale of enterprises’ imports of high-tech intermediate products; The second is to provide considerable subsidies to enterprises that import high-tech products, mobilize their enthusiasm, and absorb and learn advanced clean technology and management experience to improve the industrial pollution emission control system.

Second, broaden the channels for obtaining information on foreign intermediate products and promote the diversification of imported intermediate products. This paper has verified that the import of intermediate goods can enhance the diversification of imported products, and an increase in the variety of imported goods can promote pollution emissions in the industrial sector. Therefore, it is necessary to actively promote the transformation of active import trade from intensive marginal to expanding marginal, that is, from quantity to quality. By continuously improving relevant trade policies, deepening bilateral trade cooperation with multiple countries or regions, and accelerating the construction of a global network of high standard free trade zones, the diversity of imported intermediate goods can be enhanced. Then absorb more high-quality embedded knowledge and technology from it, promote the green upgrading of industrial production models, and accelerate the green and low-carbon transformation and high-quality development of the manufacturing industry.

Third, improve the ability to absorb and learn technology, and promote technological progress in the industrial sector. The import of intermediate goods promotes technological progress in the industry through three effective paths, which in turn affects the pollution emissions of the industrial sector. To this end, more preferential policies and financial subsidies should be implemented, while increasing research and development investment within the industry to make it easier for them to absorb and learn technology and management experience from imported intermediate products, improve the industry’s ability to absorb and learn technology, enhance independent innovation and secondary innovation capabilities, and improve production technology to reduce pollution emissions in the industry’s production process. Advanced management awareness has a certain enlightening effect on environmental pollution control to guide the industry’s awareness of environmental protection.

Fourth, import relatively inferior intermediate goods and optimize the allocation of industry factor resources. Implement differentiation strategies based on industry characteristics, and expand the import scale of resource products required for heavily polluting industrial sectors; Mild pollution industries, mainly including high-tech industry departments, should increase the import of high-tech intermediate products, and learn from the technology and management experience accompanying imported intermediate products. Promote digestion, absorption, and reinnovation, build a market-oriented green technology innovation system, implement green technology innovation initiatives, carry out resource efficiency benchmarking and improvement actions for key industries and products, and improve the technological impact and overall resource allocation efficiency of the economy.

### Research limitations and prospects

This study focuses on China, a major intermediate goods trading country, demonstrating that intermediate goods imports reduce industrial pollution emissions through import competition effect, variety effect, and technology spillover effect. However, there is still room for improvement: firstly, although it is quite representative to study Chinese industry with its large volume of imported intermediate products, for other country samples, there may be a threshold effect of technology spillovers from imported intermediate products on pollution emissions, which can be considered in future research; secondly, this study preliminarily proves that the variety effect is one of the channels of action. Future research can discuss whether the variety effect of intermediate goods imports on industrial pollution emissions is influenced by the source of the import, in order to make import policies more targeted.
